# Permutation Entropy: An Ordinal Pattern-Based Resilience Indicator for Industrial Equipment

**DOI:** 10.3390/e26110961

**Published:** 2024-11-08

**Authors:** Christian Salas, Orlando Durán, José Ignacio Vergara, Adolfo Arata

**Affiliations:** 1 Escuela de Ingeniería Mecánica, Pontificia Universidad Católica de Valparaíso, Valparaíso 2340025, Chile; christian.salas.c@mail.pucv.cl (C.S.); adolfo.arata@pucv.cl (A.A.); 2 RMES Analytics, Santiago 7560742, Chile; jose.vergara@rmesanalytics.com

**Keywords:** industrial resilience, permutation entropy, ordinal patterns, resilience assessment

## Abstract

In a highly dynamic and complex environment where risks and uncertainties are inevitable, the ability of a system to quickly recover from disturbances and maintain optimal performance is crucial for ensuring operational continuity and efficiency. In this context, resilience has become an increasingly important topic in the field of engineering and the management of productive systems. However, there is no single quantitative indicator of resilience that allows for the measurement of this characteristic in a productive system. This study proposes the use of permutation entropy of ordinal patterns in time series as an indicator of resilience in industrial equipment and systems. Based on the definition of resilience, the developed method enables precise and efficient assessment of a system’s ability to withstand and recover from disturbances. The methodology includes the identification of ordinal patterns and their analysis through the calculation of a permutation entropy indicator to characterize the dynamics of industrial systems. Case studies are presented and the results are compared with other resilience models existing in the literature, aiming to demonstrate the effectiveness of the proposed approach. The results are promising and highlight a highly applicable and simple indicator for resilience in industrial systems.

## 1. Introduction

In today’s contexts, it is crucial for an industrial system to adapt and recover from unexpected events, such as technical failures or supply interruptions, in order to maintain production and minimize economic losses. This ability of a system to withstand and recover from disturbances without significant interruption is known as industrial resilience [1]. Resilience in industrial systems is fundamental for ensuring operational continuity and efficiency in highly dynamic and disturbance-prone environments.

The increasing complexity and interconnection of modern industrial processes have sparked a growing interest in investigating and measuring the resilience of these systems [2]. Identifying and quantifying resilience can provide valuable insights for enhancing the responsiveness and adaptability of industrial systems to unforeseen changes.

This article proposes a new quantitative indicator based on the use of Shannon entropy [3] applied to ordinal patterns in time series as a measure of resilience in industrial systems. This approach will enable the precise and efficient evaluation of industrial systems’ resilience, offering a valuable tool for managing and improving operability in complex industrial environments.

The structure of this article is as follows: the next section presents a brief literature review to provide a theoretical and contextual framework for the proposal. Then, in Section 3, the methodology and the derivation of the resilience indicator based on the permutation entropy of ordinal patterns are proposed. Subsequently, in Section 4, case studies are presented and discussed as a form of validation. Finally, a discussion and conclusions sections are provided.

## 2. Theoretical Background

### 2.1. The Concept of Resilience

The concept of resilience has been defined by numerous authors, but in general, it is defined as the ability to recover and maintain functionality after failures [4]. Various disciplines have incorporated the term into their areas, ranging from ecology [5], where the term originated, to materials science [6], as well as social and urban studies [7]. In the field of engineering, the American Society of Mechanical Engineers (ASME) defines resilience as the ability of a system to withstand external and internal disturbances without interruption in its function [8]. If the function is interrupted, it must be fully recovered as quickly as possible. This definition highlights the importance of maintaining the performance of a system under stable and reliable conditions, even when adverse conditions or shocks occur. This concept contributes to the management of physical assets, where failures can have significant economic and operational consequences.

Various authors have proposed models to measure resilience. Tierney and Bruneau [9] measure the recovery time of a system after a shock. Yodo and Wang [10] propose a general framework for resilience analysis. Cholda [11] suggests a holistic evaluation. Albasrawi et al. [12] develop metrics to compare recovery strategies in cyber infrastructures. Ibrahim and Alkhraibat [13] use factors such as voltage and recovery time for microgrid systems. Attoh-Okine et al. [14] consider robustness, redundancy, and resources. Hu and Mahadevan measure the resilience of mechanical systems through reliability over time. Cai et al. relate resilience to system availability in the face of disruptive events. Durán et al. use indicators such as productivity and equipment effectiveness to propose a resilience indicator [15]. Most of these models are based on subjective and qualitative approaches, which makes their generalization difficult.

### 2.2. Ordinal Patterns

In set theory, an ordinal number describes the numerical position of an object in an ordered set, allowing for the comparison of the size and order of well-ordered sets. On the other hand, a pattern is a repeated arrangement of elements that follows a specific rule [16]. Patterns can be finite or infinite and include arithmetic, geometric, or symbolic sequences, or other variations.

Ordinal patterns are sequences of elements ordered in time series, where the relative order of the elements is more crucial for the analysis than their specific value [17]. The essence of this approach lies in comparing the relative values of the data, which allows for the study of how the data are organized in relation to each other [18].

The application of ordinal patterns is particularly beneficial in areas that require a detailed study of time series with large fluctuations, where it is necessary to characterize their behavior. This method is relevant in fields such as biomedicine (e.g., in the analysis of brain waves) [19], financial analysis [20], environmental analysis [21], and, in the context of this work, the analysis of dynamics in industrial equipment or systems to characterize their performance behavior over time.

Dynamic systems often exhibit random fluctuations in their operation and performance. Ordinal patterns offer greater robustness against system noise by focusing on the order characteristics of values rather than their specific quantification [19]. This approach allows for more precise information about the underlying trends and patterns in the time series.

There is no single method based on ordinal patterns for calculating and sizing the behavior of a time series. However, all techniques converge on the idea of generating symbols that symbolically represent the time series data. Various parameters can be employed depending on the context and specific analysis requirements, including different pattern sizes, different delays between data points, and different variations in the way data are categorized and symbolized. Regardless of the technique used, the common goal is to transform the data into ordered symbols that represent the underlying structure of the time series. These symbols enable robust analysis that is less sensitive to noise, focusing on the order of values rather than their exact magnitudes.

In the context of industrial analysis, the capacity and flexibility of this approach are crucial as they allow the adaptation of ordinal pattern techniques to the specific characteristics of the data and their fluctuations. For example, in analyzing the dynamics of the operation or performance of industrial equipment, different configurations (order and delay) can be used to better capture the system’s variations and behaviors under study.

Ordinal patterns are basically defined through two main variables: the pattern size or embedding dimension (*d*) and the delay (*τ*).

Pattern embedding dimension (*d*): The pattern dimension (*d*) represents the number of consecutive points in the time series that constitute the ordinal pattern. For example: an embedding dimension, *d* = 2. This considers only a pair of consecutive points, which may not be sufficient to capture the full structure of the data. Pattern with *d* = 3. This examines triplets of points, providing a more comprehensive view of the time series and its dynamics.

A small embedding dimension may fail to capture the system’s complexity, while a larger pattern size can be more sensitive to noise and increase the computational cost for processing, as it involves more points and a greater number of possible patterns.

The embedding delay (*τ*) is the spacing between the points compared within each ordinal pattern:

*High Delay Values*: Using high delay values involves considering points that are more separated in time to generate the ordinal patterns, which can better capture long-term dynamics. However, this might result in a loss of information about short-term dynamics, as important details could be overlooked.

*Low Delay Values*: Using low delay values means comparing points that are closer together in time, which can effectively capture short-term dynamics but may be more sensitive to noise in the data.

A high delay can help mitigate data noise, allowing for a more accurate interpretation of the ordinal patterns.

**Figure 1:**
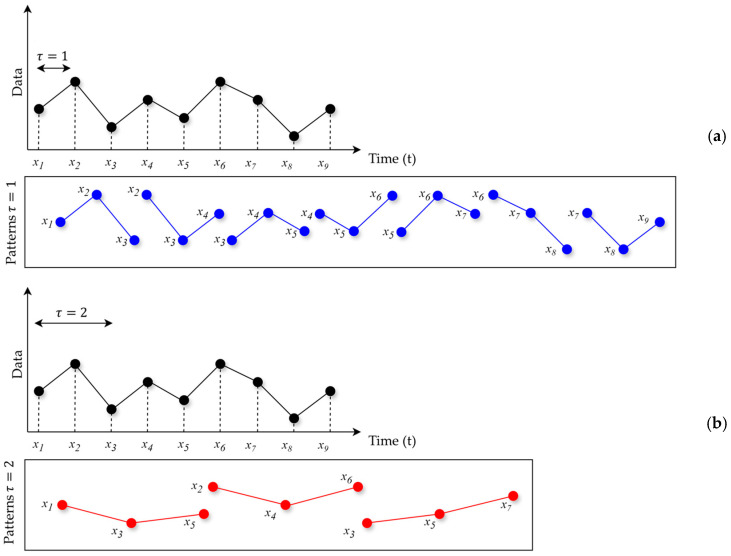
Geometry ordinal patterns. (**a**) delay equal to one in the detection of ordinal patterns. (**b**) delay equal to two.

Figure 1a shows a situation with patterns where *d* = 3 and *τ* = 1, while Figure 1b shows the situation with *d* = 3 and *τ* = 2. Note how the patterns differ with just a simple change in one of their parameters.

In general, ordinal patterns are described without considering the repetition of values, as it is unusual or unlikely for a dynamic system to exhibit exactly identical or repeated behavior in a short period of time.

This approach allows for a more refined and precise analysis of time series data, as it facilitates the differentiation of behavior patterns and their relationship to the resilience of the system under study. Additionally, it provides a solid foundation for interpreting and understanding the data in a manner relevant to the research context.

### 2.3. Permutation Entropy

Entropy is a “measure of the disorder of a system”, a concept widely known in thermodynamics [22], information theory [23], and statistical analysis. Considering industrial systems, these generally exhibit fluctuations and dynamism in their data series regarding functionality levels. Measuring the degree of “order” in these time series could indicate how resilient they are to random disturbances and how capable they are of recovering from disruptive events.

Specifically, permutation entropy is a measure of complexity for time series based on the comparison of neighboring values [24]. It is easy to calculate, robust, and useful even in the presence of noise. Permutation entropy evaluates the diversity of ordinal patterns (permutations) in a time series, with its value increasing with randomness and decreasing with predictability [25]. This measure is invariant under monotonic nonlinear transformations and is especially advantageous due to its simplicity and speed of calculation.

On the other hand, Shannon entropy is a measure of the uncertainty in a system and is calculated using the probabilities of event occurrences in a probability distribution [3,26]. This model is calculated using the following formula:

(1) $$H\left(X\right)=-\sum_{i=1}^{n}P\left(x_{i}\right)\cdot \left(P\left(x_{i}\right)\right)\;$$

where *P*(*x_i_*) is the probability of event *x_i_* occurring and *n* is the total number of possible events.

This work proposes a new resilience indicator, specifically a resilience indicator based on the permutation entropy of ordinal patterns, combining symbolic time series analysis to obtain an entropy measure that represents the time performance of the resilience of equipment or production lines.

## 3. Methodology

The proposed approach focuses on deriving a resilience indicator from a time series with values representing some measure of functionality or performance of a production system, thereby supporting the decision-making process in physical asset management. Initially, the Symbolic Analysis Method is applied to a time series containing data on the functionality of equipment or systems. This series is then discretized by dividing the space covered by the series into a finite number of elements.

Next, the symbolization process takes place, where each value corresponds to a unique symbol belonging to a finite alphabet. Thus, the time series, understood as a trajectory, becomes a finite chain of symbols. These symbols constitute the so-called ordinal patterns.

The different ordinal patterns are then identified and counted to represent changes in behavior over time in the time series. The permutation entropy method is applied to the chain of symbols in relation to the different ordinal patterns. Finally, the obtained value is evaluated and compared with entropy values derived from the proportion of certain ordinal patterns, using various series from other systems or the same system in different periods. This comparison provides information about the resilience of the analyzed system.

For the formation of ordinal patterns, a set of value ranges is considered: for example, high, medium, and low. By assigning each data point to one of these ranges, it becomes possible to identify variations in the performance or functionality of the system over time. Once the chain of patterns is constructed, a measure of permutation entropy is calculated. The main hypothesis of this work is that this measure of permutation entropy can be linked to or used to represent the resilience of the equipment or system whose performance or functionality produced the time series being analyzed.

### 3.1. Ordinal Pattern Identification

As mentioned in the previous section, the data from the time series will be classified or assigned according to ranges (e.g., high, medium, and low). To establish these ranges, the local arithmetic mean of the data is considered. The term “local arithmetic mean” refers to the fact that the time series is subdivided into a given number of constant-length segments, for which the mean is calculated.

**Figure 2:**
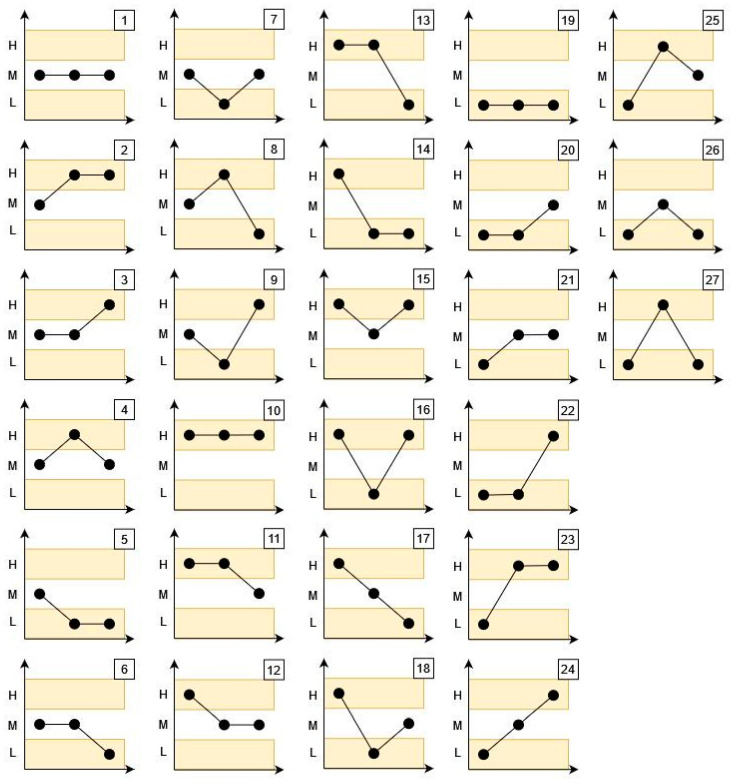
Pattern combinations.

The symbolization phase is carried out according to the number of labels considered in the analysis. For example, if the labels are high, medium, and low, the values will be classified as “high” if they are greater than the mean plus a user-defined value *l*. Values will be considered “medium” if they fall within a range around the mean +/− the value of *l*, and “low” if they are less than the mean minus *l*. Once the series values are classified, ordinal patterns are identified using combinations of these labels (Figure 2). The number of possible patterns is defined by *N_P_ = k^d^*, where *d* is the pattern order and *k* is the number of labels considered. For the analysis of ordinal patterns, patterns with a fixed size of *d* = 3 were selected, resulting in 27 possible patterns (with a length of 3) containing three nodes each. This choice was based on the theory suggesting that, with this length [19], the data structure can be robustly interpreted without generating an excessive number of combinations.

Each combination will be associated with a number between 1 and 27 to facilitate its identification and analysis in the algorithm. This approach enables a more precise analysis of time series, capturing significant variations and their relationship to the system’s resilience.

### 3.2. Definition of Lookback

As previously mentioned, the time series is segmented into uniform-sized segments, called lookbacks. Each segment is used to identify ordinal patterns, calculate the mean value of the data within the segment, establish ranges for labeling, and perform symbolic analysis. This allows for the individual analysis of resilience corresponding to each time segment. The use of local parameters in each segment captures specific variations in the system and provides a detailed view of how the system’s resilience changes over time.

The calculation of entropy assumes that each pattern affects the results of the equation equally, depending on its frequency in the time series. However, for the model to indicate the resilient behavior of a system, certain patterns should be weighted more heavily than others.

In summary, in a time series that shows a situation of greater resilience, patterns that demonstrate greater stability, less severe drops, and faster recoveries should have a higher weight than other patterns, positively impacting the resilience measure. Conversely, patterns associated with sharp drops in performance and slower recoveries are considered to negatively impact the resilience value.

Therefore, a resilience factor is added to the entropy model for each possible pattern, modifying the entropy formula as shown in Equation (2).

(2) $$\rho \left(x_{i}\right)=1-\left(\frac{1}{{log}_{2}\left(k^{d}\right)}\cdot \sum_{i=1}^{n}\frac{fr_{i}}{max(fr_{i})}\cdot P\left(x_{i}\right)\cdot \left(P\left(x_{i}\right)\right)\;\right)$$

where the term $\frac{fr_{i}}{max\;(fr_{i})}$ corresponds to the normalized resilience factors. The results obtained by applying Equation (2) will range between 0 and 1, where the system will be more resilient as the value approaches 1. Conversely, it will be less resilient as the value of ρ(*x_i_*) approaches 0. The detailed differentiation of the weights or ponderations that certain patterns will possess is analyzed in the following section.

This approach ensures that the resilience measure accurately reflects the system’s performance stability and recovery capability by giving higher weights to patterns that indicate resilience and lower weights to those indicating vulnerability.

### 3.3. Resilience Factors

**Figure 3:**
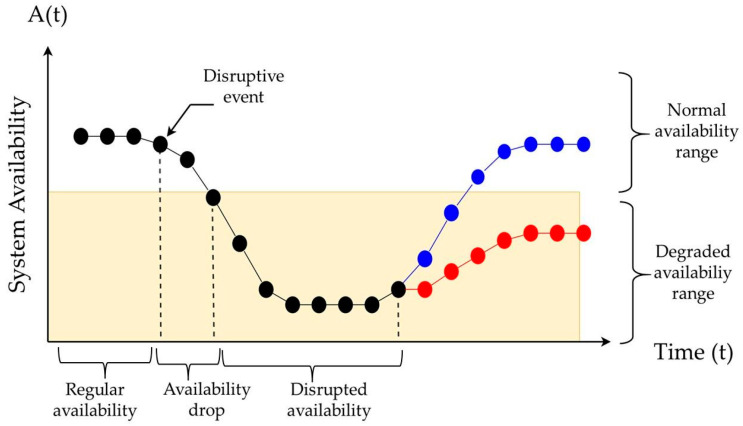
System performance after a shock.

It is crucial to conceptually analyze the transitions that patterns can present throughout the series and the need to assign different weights to each possible pattern. To do this, we will explore the relationship between resilience and disruptive events. The different scenarios that can arise from a disruptive event and may affect its performance (e.g., availability) are illustrated by Figure 3. Disruption means “a sudden break or interruption”, so a disruptive event, in the industrial context, is an occurrence that causes an interruption or loss of the system’s normal performance.

In a system that is in a stable or steady state and operates within a normal range, a sudden and disruptive event can cause a “drop” in performance, leading to a degraded performance range. In this situation, the system can exhibit two possible behaviors: (*i*) the blue segment shows that from a degraded performance level, the system returns to the normal performance level, which indicates a resilient system; or (*ii*) the system fails to recover its performance level and remains degraded. The latter indicates low resilience. If we examine in more detail what happens during a disruptive event, and by observing Figure 3, we can identify the following phases:

1. Normal Performance: In this stage, the system operates regularly with an availability *A*_0_.
2. Performance Drop: Due to a disruptive event, the system degrades from *A*_0_ to *A_D_* between times *t_I_* and *t_II_*.
3. Disrupted Performance: The system stabilizes at a lower-than-acceptable availability level, *A_L_*. During this stage, corrective and maintenance actions are applied to begin recovering the system’s performance at time *t_III_*.
4. Recovery: Due to the recovery efforts, the system’s availability increases, surpassing the acceptable availability limit and reaching an acceptable level by time *t_IV_*.
5. Recovered Performance: The system returns to normal operation, regaining its initial availability or something close to *A*_0_.

Each node of the pattern locally represents the stage the system is in. This characteristic is crucial in defining how ordinal patterns affect resilience.

Given that the nature of ordinal patterns does not consider the temporal dimension, it is important to conceptually define which stages and conditions in the system are more resilient. Ordinal patterns only consider the relative order among them, so assigning them a weight is a way to relate the transitions to their respective timeframes, the stage they represent, and the performance indicated by the pattern. Without these adaptations, entropy would only measure the degree of disorder within the segments of the series, without directly relating to the concepts of resilience. By linking the transitions to the times and local data of each pattern, an important question arises: Which patterns most influence the resilience of the system? To propose a unique set of weights, we will focus primarily on the parameters (*A*_0_ *− A_D_*) and (*t_III_ − t_II_*), which represent the performance drop after a disruptive event, and the time spent in a disrupted state or the time it takes for the system to begin its recovery.

Using the definition provided by ASME, a resilient system must be able to recover its functions quickly. This indicates that (*t_III_ − t_II_*) should be as short as possible, regardless of the magnitude of the system’s availability drop.

**Figure 4:**
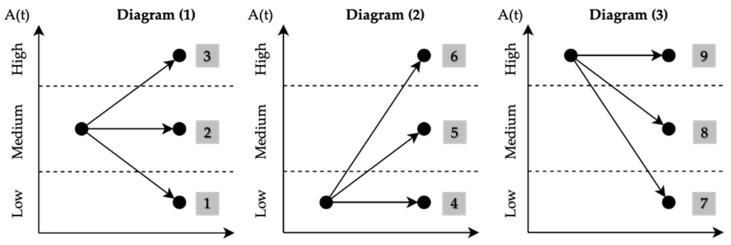
Transition diagrams. Where nodes represent the specific states of the system, arrows indicate the direction and type of transition between states. The numbers next to the arrows indicate the type of transition according to the classification in the analysis.

To facilitate understanding of the combinations of different patterns, transition diagrams will be used to describe these state transitions. These diagrams show the possible transitions in the performance *P*(*t*) of a system over time *t*, starting from an initial state *P*_0_. Each transition is labeled with numbers representing different scenarios of performance change (Figure 4).

The patterns are differentiated by two main characteristics related to the transitions they present intra-pattern. Firstly, different importance is assigned to transition 1 ($T_{1i}$) and transition 2 ($T_{2i}$), prioritizing which range the final node falls into. This is because the final state of a system after a disruptive event is crucial for determining its future performance. Assigning greater importance to the last node allows for a more accurate reflection of the impact on the system’s resilience. Additionally, this priority also reflects the system’s stability, as the last node indicates the range in which the system’s performance has stabilized.

As mentioned earlier, the different ordinal patterns have specific weights reflected in the proposed resilience equation. These weights are determined by their contribution to the system’s resilience. In other words, patterns that positively influence the system receive a weighting that enhances resilience, while patterns that negatively affect resilience receive a weighting that reduces it.

The resilience factors represent the weight of each pattern in the resilience calculation and are determined using the following equation:

(3) $$fr_{i}=1+(\alpha \cdot T_{1i})+(\beta \cdot T_{2i})$$

where

▪ *i* is the pattern number between 1 and 27.
▪ $T_{1i}$ is the contribution of the first transition between nodes $N_{1}$ and $N_{2}$.
▪ $T_{2i}$ is the contribution of the second transition between nodes $N_{2}$ and $N_{3}$.
▪ α and β are importance factors that modify the weight of the transitions intra-pattern and depending on how the pattern ends.

The importance factors modify the weighting of the pattern transitions depending on transition $T_{2i}$, as this last transition more significantly reflects the nature of the pattern and its final contribution to the representation of the system’s resilience, as reflected in the time series. Thus, as a result of various sensitivity analyses, the values of *α* and *β* were defined according to the following relationship: *α* = 0.75 *β*. Since the method is parameterizable, the value of Beta can be predefined by the user, just like the values of the ranges. In our case, we used a Beta value of 1.

The contribution values according to the type of intra-pattern transition are represented by the value assigned to the transition ($T_{1i}$ and/or $T_{2i}$). To this end and considering the 9 types of transitions shown in Figure 4, the values that each type of transition will have for the calculation of the *f_ri_* according to Equation (3) were established in collaboration with a group of maintenance experts, operating under a focus group modality. These values, along with a brief explanation for each one based on the type of transition, are shown in Table 1.

The resilience factors are multiplied by the probability in the entropy equation, representing the contribution of each pattern to the system’s ability to resist and recover from disturbances. The contribution percentages were selected to reflect the impact of each type of transition on the system’s resilience:

A. Improvement, Stable Recovery, Enhanced Recovery: These transitions indicate that the system is improving its performance or recovering after a disturbance, thereby increasing resilience.

B. Disruptive Stages, Stable Disruptive, Severe Disruptive: These transitions represent a significant reduction in the system’s performance, thereby decreasing resilience.

C. Stability, Stable Improvement, Normalization: These stages reflect that the system remains within an expected performance range or improves slightly without significant changes in resilience.

## 4. Application Example

To demonstrate the application of the model and validate the use of ordinal patterns and permutation entropy as a reliable measure of resilience, a synthetic series containing 50 data points on equipment availability was used. This series was divided into 5 segments, each containing 10 consecutive data points. These segments were selected to represent different states or typical behaviors, both in terms of their magnitudes and variability. The idea was to provide a set of values that would shed light on the discussion of how well the proposed resilience indicator can represent a qualitative description of a system’s resilient behavior. The configuration of the proposed model was chosen for its simplicity:

▪ τ=1
▪ *Lookback* = 10

**Figure 5:**
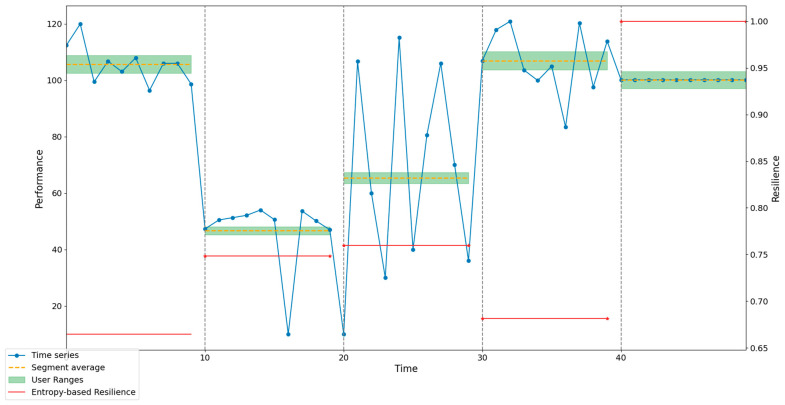
Resilience, ranges, and mean per segment, τ=1 and *lookback* = 10.

**Figure 6:**
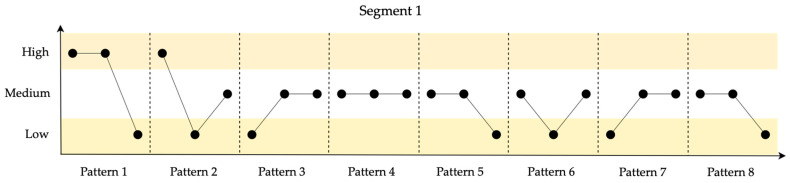
Identified patterns for the 10 data points in Segment 1.

As part of the symbolization procedure, and in order to facilitate the subsequent qualitative analysis of the example, the time series was subdivided into 5 segments. Additionally, each segment was assigned its own range, considering +/−3% of the mean value of the data in each segment, thereby establishing the boundaries for the low, medium, and high ranges. Subsequently, patterns were identified in each segment. Permutation entropy was then calculated using 8 sliding time windows of constant size (3) for each segment. Figure 5 illustrates the series, the respective ranges for the symbolization process, the mean value of each segment, and the corresponding resilience value. To enhance understanding of the proposed model, a detailed analysis and discussion of each segment in the series is provided. Figure 6 shows the identified patterns for the 10 data points in Segment 1.

**Figure 7:**
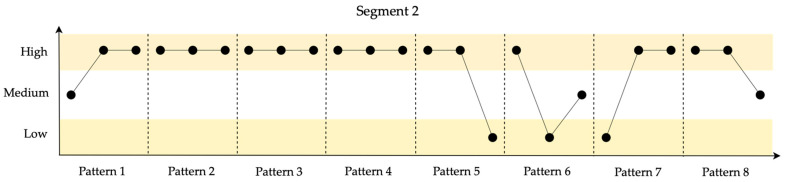
Identified patterns for the 10 data points in Segment 2.

In Figure 7, the identified patterns for the 10 data points in Segment 2 are shown.

**Figure 8:**
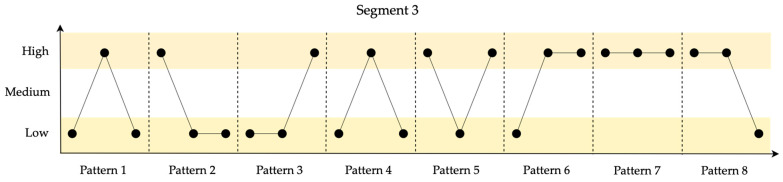
Identified patterns for the 10 data points in Segment 3.

In Figure 8, the identified patterns for the 10 data points in Segment 3 are shown.

**Figure 9:**
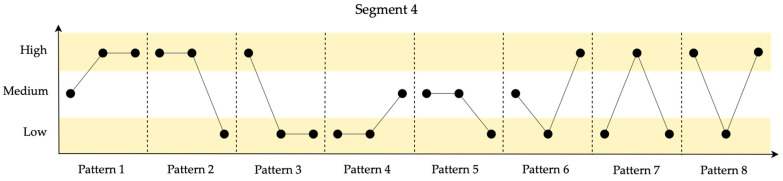
Identified patterns for the 10 data points in Segment 4.

In Figure 9, the identified patterns for the 10 data points in Segment 4 are shown.

**Figure 10:**
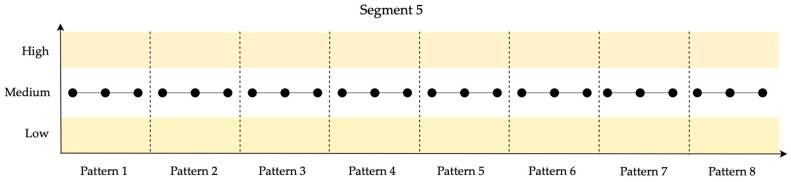
Identified patterns for the 10 data points in Segment 5.

In Figure 10, the identified patterns for the 10 data points in Segment 5 are shown.

Table 2 presents the identifiers (IDs) along with the frequency with which they were detected in each segment, representing the patterns and their frequencies of occurrence within each segment (the meaning of each pattern ID is provided in Figure 2). Additionally, the table includes the mean values of the data within each segment, their standard deviation, and the resilience value calculated for each time segment, as determined by Equation (1).

Segment 1 demonstrates moderate resilience despite initial impressions of good performance stability. This is attributed to different ordinal patterns with lower relative frequencies, indicating unstable behavior.

Segment 2 exhibits superior resilience due to two factors: relatively stable performance values with fewer distinct patterns and the system’s quick recovery to a stable range following a disruptive event. This rapid recovery reflects high resilience.

Segment 3 shows the lowest resilience, primarily due to the use of permutation entropy, where frequent patterns correlate with better resilience.

Segment 4, while not having predominantly negative patterns, lacks stable trends and shows considerable data variability. Consequently, it can be interpreted as less resilient compared to other segments.

Finally, Segment 5 maintains complete stability, representing the highest resilience level, characterized by a single pattern (pattern 1) with a frequency of 8, indicating no fluctuations in the data.

By examining this illustrative example, it can be concluded that the resilience value does not depend on the magnitude of the values used for its calculation, but rather on the relationships between the values or symbols considered. In other words, resilience is a measure of stability derived from the behavior of a dataset. Furthermore, if the entropy value is adjusted by giving greater weight to certain ordinal patterns that better represent the behavior or reaction to a system’s or entity’s performance drop, the altered entropy value will be closely associated with the characteristic intended to be extracted from the data—resilience.

## 5. Case Study

**Figure 11:**
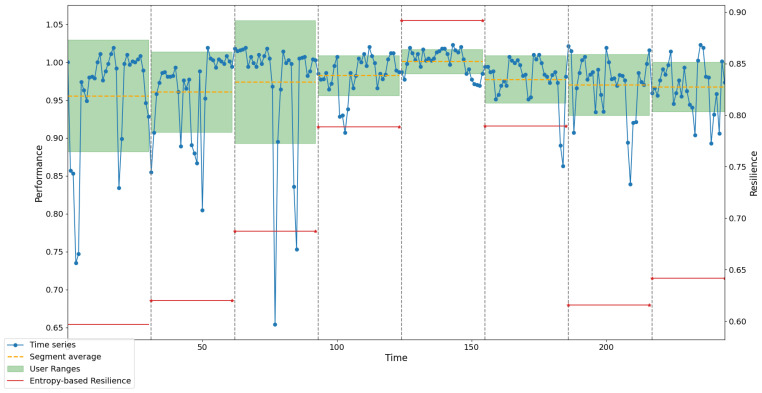
Resilience, ranges, and mean per segment of the actual time series.

In order to validate and demonstrate the utility of both, the model and the proposed methodological procedure, an analysis was conducted on the resilience index behavior of a fleet used in the operation and exploitation of an open pit mine located in northern Chile. This fleet consists of 4 electric shovels, 5 drills, and 42 trucks, comprising 51 units. Support equipment, such as motor graders and water trucks, among others, were excluded. Historical availability data at the fleet level were collected, covering a continuous 244-week period of operation. Subsequently, this series underwent the symbolization process, as described in the previous section. Once the values were replaced by the corresponding symbols, ordinal patterns were identified using sliding windows and subsequently counted. Finally, the resilience values were calculated using the proposed procedure. The data series, as well as the value ranges for the low, medium, and high intervals for each segment, averages, and the resilience value for each segment, are shown in Figure 11.

As mentioned earlier, there are several methods in the literature for quantifying the resilience of industrial systems. To compare the results provided by the proposed model (Method A), two existing resilience indicators from the literature were computed.

The availability-based resilience method, known as the “Availability-based engineering resilience metric”, evaluates a system’s ability to maintain and recover its functionality after disturbances. It measures the system’s availability in a steady state before and after external shocks, as well as the time required to reach these states. Equation (4) computes a resilience value through the integration of availability values and times that reflects both the system’s performance and recovery processes [27]. This method will be referred to as Method B.

(4) $$\rho =\left(\frac{A_{1}}{ln\;\left(t_{1}\right)\;}\right)\cdot \sum_{i=1}^{n}\left(\frac{A_{i2}{\;\cdot A}_{i3}}{ln\;\left(t_{i3}-t_{i2}\right)\;}\right)$$

where

▪ $A_{1}$ is the system’s availability in a steady state before the shock.
▪ $t_{1}$ is the time required to reach the initial steady state.
▪ $A_{i2}$ is the transient availability of the system immediately after shock i.
▪ $A_{i3}$ is the system’s availability in the new steady state after shock i.
▪ $t_{i2}$ and $t_{i3}$ are the times corresponding to the states $A_{i2}$ and $A_{i3}$, respectively.

The resilience method based on performance and recovery, described by Cheng et al. [28,29] measures a system’s ability to resist, adapt to, and recover from disturbances. It evaluates the system’s performance during periods of decline and recovery, as well as the time needed to restore its performance. The resilience metric integrates performance across different periods and the speed of recovery, providing a quantitative assessment of the system’s robustness and recovery capability. This method will be referred to as Method C.

(5) $$\rho \left(t_{x};t_{m}<t_{x}\leq t_{m+1}\right)=1+\sum_{j=1}^{m-1}\sum_{i=1}^{t_{j+1}-t_{j}}\frac{P_{norm}\left(t_{j}\left(i\right)\right)-P_{norm}\left(t_{j}\left(i-1\right)\right)}{t_{{norm}_{j}}\left(i\right)-t_{{norm}_{j}}\left(i-1\right)}+\sum_{i=1}^{t_{x}-t_{m}}\frac{P_{norm}\left(t_{m}\left(i\right)\right)-P_{norm}\left(t_{m}\left(i-1\right)\right)}{t_{{norm}_{m}}\left(i\right)-t_{{norm}_{m}}\left(i-1\right)}$$

where

▪ $P_{norm}\left(t_{j}\left(i\right)\right)$ is the system’s normalized performance over time $t_{j}\left(i\right)$.
▪ $t_{{norm}_{m}}$ is the normalized duration time of segment m.

The values obtained by each method for the nine segments are shown in Table 3.

To compare the resilience values between the studied indicators, two different methods were employed:

- Coefficient of Determination (*R*^2^): This method measures how well the observed outcomes are replicated by the model, indicating the proportion of the variance in the dependent variable that is predictable from the independent variable.
- Bland–Altman Model: This approach evaluates the agreement between two measurement methods by plotting the differences between the measures against their mean, allowing for the identification of any systematic bias [30].

**Figure 12:**
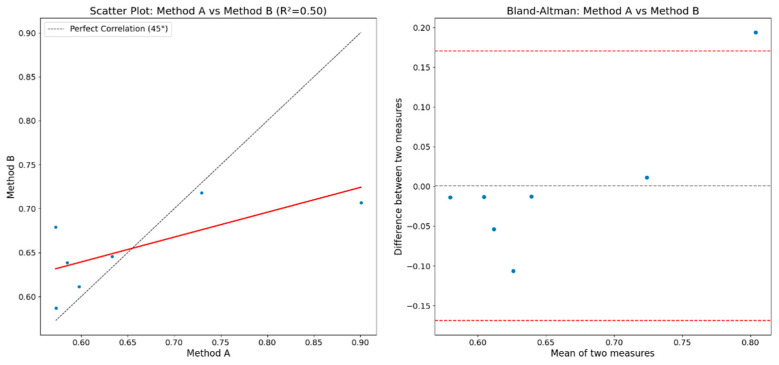
Correlation and Bland–Altman plots with values obtained by Methods A and B.

Figure 12 shows the correlation plot and the Bland–Altman plot for comparing the results obtained between Method A and Method B. It can be observed that the coefficient of determination (*R*^2^) has a value of 0.50, which is interpreted as a moderate correlation between the methods.

**Figure 13:**
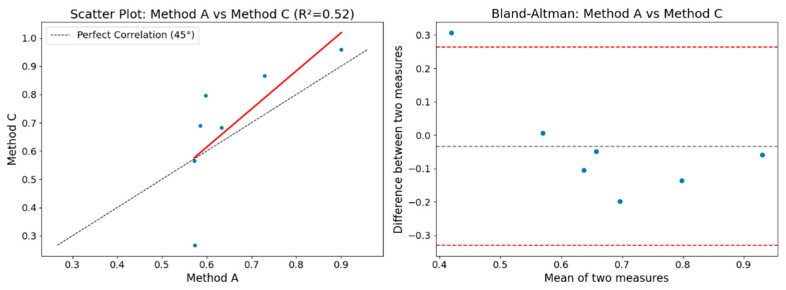
Correlation and Bland–Altman plots with values obtained by Methods A and C.

Figure 13 shows the correlation plot and the Bland–Altman plot for comparing the results obtained between Method A and Method C. A correlation coefficient of 0.52 is observed, indicating a moderate correlation.

When observing both Bland–Altman plots, almost all points are within the variability interval limits. This means that the differences between the measurements of the three methods are within an acceptable range of variability. This implies a certain and good consistency between the methods, with no extreme differences or significant disagreements among the three methods. This also indicates the absence of systematic bias that could cause major discrepancies.

**Figure 14:**
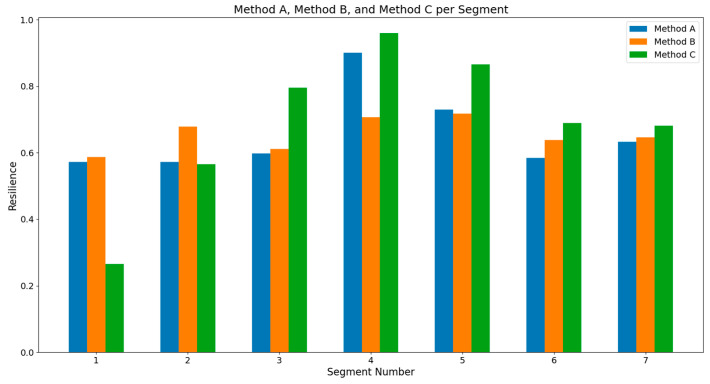
Resilience values obtained by the three methods under comparison.

Figure 14 shows the comparison between the resilience values obtained by the three methods. As observed, there is a relative similarity in the behavior of the values. Segments 1 to 8 were used because Segment 9, not having the same number of periods as the previous segments, distorts the resilience values and does not allow for correct comparison. As previously mentioned, there is no complete agreement between the resilience values calculated by the three methods. However, a relatively similar behavior can be observed among them. When considering the two methods cited in the literature, the same discrepancy is evident. This suggests that the field remains open and that no universal resilience indicator exists, hence the proposal of this new resilience indicator based on the permutation entropy of ordinal patterns.

Ultimately, these analyses and comparisons lead to the conclusion that, when compared to two other established methods in the literature, the resilience measurement approach based on the permutation entropy of ordinal patterns is both reliable and viable as an alternative, offering an acceptable level of accuracy.

## 6. Conclusions

In this work, we have presented a proposal for a new resilience indicator based on the application of permutation entropy of ordinal patterns to a time series transformed into a symbolic series. This approach has demonstrated its applicability and feasibility through a practical case study.

This innovative methodology offers a robust approach under conditions of noise and variability, making it a valuable tool for resilience studies in various fields. The application of permutation entropy based on ordinal patterns and the transformation into symbolic series allows for fast and accurate calculations, facilitating the real-time analysis of large datasets. Thus, this proposal constitutes a computationally efficient method for evaluating resilience in engineering systems, enabling real-time analysis of large datasets. Additionally, the method is flexible enough to adapt to different types of time series and systems. Along with this, the method is parameterizable, as it allows for the adjustment of its key parameters to adapt to specific situations, such as production cycles, new maintenance strategies, etc. In summary, the proposed indicator represents a contribution in the field of resilience quantification, offering a new perspective for examining and understanding complex systems.

The managerial insights derived from this work offer guidance for improving operational efficiency and long-term sustainability. One key takeaway is the influence of maintenance strategies on resilience indicators. Proactive maintenance approaches, such as predictive and preventive strategies, can significantly enhance system resilience by minimizing unplanned downtimes and ensuring more stable operations. In contrast, reactive maintenance may lead to greater variability and reduced resilience, as it often fails to address potential failures in a timely manner. The challenge for managers lies in translating these insights into actionable practices, requiring an alignment of resources, employee training, and technological integration to implement data-driven maintenance strategies. Efforts to embed resilience measurement into the operational workflow will involve not only adjusting existing processes but also fostering a culture of continuous improvement and responsiveness to system feedback. Ultimately, adopting and integrating resilience measurement tools and metrics will provide managers with real-time insights that enable more informed decision-making, optimizing both performance and adaptability in dynamic production environments.

Future work will focus primarily on continuing the validation of this method in different applications and on the continuous improvement of the underlying algorithm. We plan to use larger ordinal patterns and test this strategy in more extensive time series. Additionally, we will continue to compare this new resilience indicator with other indicators or values obtained thorough different methodologies to evaluate its relative performance.

## Figures and Tables

**Table 1:** Contribution values according to the type of transition.

Transition	Stage Name	Description	Contribution
1	Moderate drop	Performance reduction due to a disruptive event.	5
2	Stable at a medium level	The system operates within the expected range without significant changes in performance.	3
3	Sharp rise	The system’s performance improves.	2
4	Stable at a low level	After a disruptive event, the system stabilizes at a vulnerable performance level.	4
5	Gradual recovery	The system recovers to an expected performance state.	2
6	Rapid recovery	The system not only recovers but reaches a superior performance level.	1
7	Disruptive drop	Significant performance reduction from a high state to a vulnerable state.	5
8	Moderate decline	Performance reduces to a medium or expected level.	3
9	Stable at a high level	Performance improves and maintains at a high level.	1

**Table 2:** Detected patterns and characteristic values of each segment.

Segment	Patterns [Pattern ID: Frequency]	Average	Standard Deviation	Resilience
1	{13: 1, 18: 1, 21: 2, 1: 1, 6: 2, 7: 1}	105,660	6.606	0.635
2	{2: 1, 10: 3, 13: 1, 16: 1, 23: 1, 11: 1}	46,662	12.413	0.691
3	{27: 2, 14: 1, 22: 1, 16: 1, 23: 1, 10: 1, 13: 1}	65,439	34.551	0.631
4	{2: 1, 13: 1, 14: 1, 20: 1, 26: 1, 9: 1, 27: 1, 16: 1}	106,941	11.168	0.592
5	{1: 8}	100,161	0.000	1.000

**Table 3:** Resilience values for each segment.

Segment	Method A	Method B	Method C
1	0.573	0.587	0.266
2	0.573	0.679	0.566
3	0.598	0.611	0.796
4	0.901	0.707	0.960
5	0.729	0.718	0.866
6	0.585	0.639	0.689
7	0.633	0.646	0.682
8	0.573	0.587	0.266

## Data Availability

Data are unavailable due to privacy.
